# Patient Satisfaction of Telemedicine in Pediatric and Young Adult Type 1 Diabetes Patients During Covid-19 Pandemic

**DOI:** 10.3389/fpubh.2022.857561

**Published:** 2022-03-22

**Authors:** Marta Bassi, Marina Francesca Strati, Stefano Parodi, Simone Lightwood, Clara Rebora, Francesca Rizza, Giuseppe d'Annunzio, Nicola Minuto, Mohamad Maghnie

**Affiliations:** ^1^Department of Neuroscience, Rehabilitation, Ophthalmology, Genetics, Maternal and Child Health, University of Genoa, Genoa, Italy; ^2^Department of Pediatrics, Istituto Giannina Gaslini, Genoa, Italy; ^3^Epidemiology and Biostatistics Unit, Istituto Giannina Gaslini, Genoa, Italy; ^4^IT Service (Servizio Informatico Aziendale-SIA), Istituto Giannina Gaslini, Genoa, Italy

**Keywords:** telemedicine, telenursing, type 1 diabetes, continuous glucose monitoring, insulin pump, COVID-19

## Abstract

The aim of this study was to evaluate the satisfaction of the use of telemedicine and telenursing in children and young adults with Type 1 Diabetes and their families followed in the Regional Pediatric Diabetes Center of Giannina Gaslini Institute (Liguria, Italy). An anonymous survey form was administered to 290 patients (138 filled out by caregivers and 152 by patients). The questionnaire consisted of two parts: the first one included a series of questions related to the patient's personal and medical data; the second one was directed toward the satisfaction in the use of telemedicine and telenursing during Covid-19 pandemic. The data collected showed that 92.4% of the population was overall very satisfied with the quality of the service provided. Satisfaction was much higher especially in those who live outside of the province of Genoa (*p* = 0.017) and in those on insulin pump treatment (*p* = 0.037). Telemedicine and telenursing have an essential role in diabetology and are highly appreciated in our Center, where most patients prefer to continue regular follow-up via video-call as well as in person. Telenursing was also proved to be an effective and appreciated tool for educating and supporting patients using insulin pumps and glucose sensors.

## Introduction

The Covid-19 pandemic is a global health emergency that has highlighted the need for an innovative technological system to be able to take care of patients with chronic diseases (1, 2). Digital transformation was already underway, especially in the field of diabetes, but this global emergency has accelerated this phenomenon. A large number of patients were unable or unwilling to participate in routine follow-up visits in person and strict infection prevention protocols were established; therefore, many diabetes centers have begun to rely more on virtual health care (3). Diabetes more than other areas is able to pioneer technological advances in medicine thanks to the use of many tools such as continuous glucose monitoring (CGM) and continuous subcutaneous insulin infusion (CSII) connected to online platforms for sharing data; therefore, patients are always able to share glucose data with healthcare professionals and eventually modify insulin treatment if required (4).

Although the term telemedicine does not have a unique definition in the literature, the World Health Organization and the Italian Ministry of Health has defined telemedicine as a means of providing health care services, through the use of innovative technologies, in situations where the health professional and the patient are not in the same location (5, 6). Telenursing is considered a part of telemedicine that focuses on the delivery and management of care services using telecommunication technology in the nursing field (7, 8).

In Italy, many Pediatric Diabetes Centers implemented and intensified the regular use of telemedicine during the pandemic, with a gradual increase in the official recognition of the service and the consequent reimbursement to the patient by the national health system (9, 10).

The Pediatric Diabetes Center of Giannina Gaslini Institute is an Academic Regional reference center for Liguria, Italy. In this region, after an initial period of “self-made telemedicine” (Skype televisits), “Diabetes Televisit” was officially recognized as a health intervention, available through the use of company and regional platforms and adequately reimbursed, starting from January 1st 2021. Before Covid pandemic we used online data sharing platforms, emails and cell phone communications with patients in our center, but we did not use televisits until March 2020, when we started to use telehealth regularly in order to make outpatient visits. We have also used hospital informatic platforms for the education of patients' families at the time of diabetes onset, for meetings between healthcare professionals and schools, for education on carbohydrate counting with our dietician and for psychological support with our psychologist. We have also started a telenursing service for video call support during procedures related to the use of infusion pumps (such as sensor or infusion set insertion and change).

Most of the studies available in the literature reported high patient satisfaction in using telemedicine during the Covid-19 pandemic (11). In pediatrics, telemedicine has been used for a long time (12) and during the pandemic many studies have reported the usefulness and the satisfaction of patients in the use of this tool in various fields such as rheumatology, surgery, emergency care, pneumology, and haemato-oncology (13–17).

In diabetic patients, satisfaction of telemedicine was primarily assessed in patients with Type 2 diabetes or in adults with Type 1 diabetes (T1D). Scott et al.' studies evaluating the satisfaction of T1D patients do not focus exclusively on the pediatric population and report data relating to telemedicine carried out more in the “phone call” mode rather than “video call” mode (18–20). A recent study also shows how Diabetes nurse practitioners who used telenursing during Covid-19 pandemic improved their professional status, acquired new skills and were satisfied with their personal and professional growth (21).

The aim of this study was to evaluate the impact of telemedicine and telenursing in pediatric and young patients with T1D and their families and the level of satisfaction with their use.

## Materials and Methods

The primary aim of the study was to evaluate the satisfaction in using telemedicine and telenursing of pediatric and young patients and their families followed by the Regional Pediatric Diabetes Center of Giannina Gaslini Institute, Genoa, Liguria, Italy.

All patients diagnosed with Type 1 diabetes according to ADA criteria and their parents who agreed to participate in the data collection and who gave informed consent were involved in the study. Patients and caregivers who were unable to understand, read or write in Italian were excluded.

A survey was administered anonymously. One individual per family answered the questionnaire: a parent or the patient based on the age or the child's level of independence. The first part of the questionnaire consisted of a series of questions related to the patient's personal and medical data including date of birth, age, sex, age at disease onset, city of residence, type of insulin therapy (insulin pump—CSII—or multiple daily injection—MDI), use of continuous glucose monitoring (CGM), participation in therapeutic educational camps, number of televisits (video call mode) and telenursing carried out.

The second part of the questionnaire was created starting from the questionnaire used by Palandri et al. to evaluate the satisfaction of pediatric patients in the use of telemedicine in haemato-oncology during the Covid-19 pandemic (17). We have revised and modified some items to better adapt the questionnaire to the diabetes area. We maintained three clusters from the original questionnaire: Cluster A—Adequacy of medical care, Cluster B—Psychological impact of telemedicine and Cluster C—Possible advantages and future use of telemedicine. Cluster D (Adequacy of the informatics system in original questionnaire) has been replaced with Telenursing (Table 1).

**Table 1:** Survey—second part: evaluation of satisfaction.

	**Question**	**Score: 0–6**	**Score: 7–10**
		***N* (%)**	***N* (%)**
Cluster A—Adequacy of Medical care	I was able to explain my medical problems well enough via televisit	26 (10.1)	231 (89.9)
	The absence of physical contact during the televisit was not a relevant problem	66 (25.9)	189 (74.1)
	Overall, I am satisfied with the quality of the service provided via televisit	9 (7.6)	109 (92.4)
Cluster B—Psychological impact of telemedicine	I was easily able to talk with the medical team during the televisit	23 (8.9)	234 (91.9)
	I felt at ease when communicating with my medical team	26 (10.2)	228 (89.8)
	I received adequate attention	13 (5.1)	242 (94.9)
	I perceived telemedicine as an attention toward me in this period	36 (14.2)	217 (85.8)
Cluster C—Possible advantages and future use of telemedicine	I think that televisits are an adequate modality of assistance for my disease	82 (31.9)	175 (68.1)
	I am willing to continue some of my follow-up visits via videocall, keeping appointments in person at longer intervals	51 (19.9)	205 (80.1)
	Televisits allow me to save time/money and/or time off work **(only for parents)**	26 (23)	87 (77)
		**Yes**	**No**
Cluster D—Telenursing	Did you place/change an insulin pump after February 2020?	112 (41.95)	155 (58.05)
	(**If yes q.11**) Did you change your first infusion set via televisit?	36 (32.1)	76 (67.9)
		**Score: 0–6**	**Score: 7–10**
		***N*** **(%)**	***N*** **(%)**
	**(If yes q.12)** The nurse's support is effective during telenursing	0 (0)	36 (100)
	**(If yes q.12)** I wouldn't need more telenursing appointments	8 (22.2)	28 (77.8)

Before the administration, the comprehension and the complexity of the questionnaire was assessed, and then modified by 10 healthcare professionals and 10 patients/families during medical visits in the Endocrinology and Diabetology Centers of Giannina Gaslini Institute.

To follow the same development criteria of the original questionnaire, it was decided to highlight the level of satisfaction of patients/parents thanks to a scale of points from 0 to 10 (0 = extremely unsatisfied and 10 = extremely satisfied), subsequently divided into two sections: the first going from 0 to 6 (neutral) and the second from 7 to 10 (good-great). The Cluster D—Telenursing was based on some multiple choice questions (Yes or No) and some with satisfaction scale, the latter dedicated to those who actually used the telenursing service.

The answers were also compared based on the age of the patient, the type of participant (parent/caregiver or patient) and personal or clinical characteristics reported in Table 2.

**Table 2:** Clinical and personal characteristics of 290 patients who participated to the study.

**Residency**	***N* (%)**
Genoa	198 (68.3)
Liguria (excluding province of Genoa)	53 (18.3)
Other	39 (13.4)
**Type of insulin therapy**	
Multiple daily injections (MDI)	74 (25.5)
Insulin pump (CSII)	216 (74.5)
**Use of glycemic sensor**	
Yes	268 (92.4)
No	22 (7.6)
**Number of televisits (Feb 2020–Sep 2021)**	
None	29 (10)
1–2	106 (36.6)
>3	155 (53.4)
**Number of camps attended**	
None	85 (29.3)
1–2	115 (39.7)
>3	90 (31)

Questionnaire administration, data collection and subsequent analysis were conducted from June 2021 to September 2021.

### Statistical Methods

The quantitative variables have been described through the estimate of mean and standard deviation, whereas the qualitative variables were evaluated calculating absolute and percentage frequency. The comparison between the answers to the items in the questionnaire in the different groups of interest was made by means of Pearson's chi-squared test or Fisher's exact test when appropriate, that is in the presence of a number of events expected lower than 5 in at least one cell. For every comparison, a *p*-value inferior to 0.05 was considered statistically significant. All the analyses were performed with the following software: STATA for Windows Version 13.1 (STATA Corporation, College Station, TX, USA).

## Results

We collected 290 questionnaires: 138 (47.6%) were filled out by parents or caregivers and 152 (52.4%) by the patients. Considering the whole study population, the mean (SDS) age of patients was 17.9 years. Considering only those who answered the questionnaire, the mean (SDS) age of the patients was 21.2 years (*N* 152, 30.9% <18 years of age), the mean (SDS) age of the parents/caregivers was 46.8 years (*N* 138) and the mean (SDS) age of the patients for whom the parents answered the questionnaire was 12.9 years (*N* 138). The personal and health details of the population are summarized in Table 2.

Most of the patients lived in Genoa (68.3%), 18.3% in the remaining areas of the Liguria region and 13.4% in other Italian regions. The majority of patients used glucose sensor (92.4%) or insulin pump (74.5%). Only 29.3% of patients had never participated in therapeutic educational camps. As far as the use of telemedicine, most of the patients carried out more than three televisits during the pandemic period and only 10% had never used telemedicine: those who have not used the telemedicine service we have offered did not complete the second part of the questionnaire.

**Figure 1:**
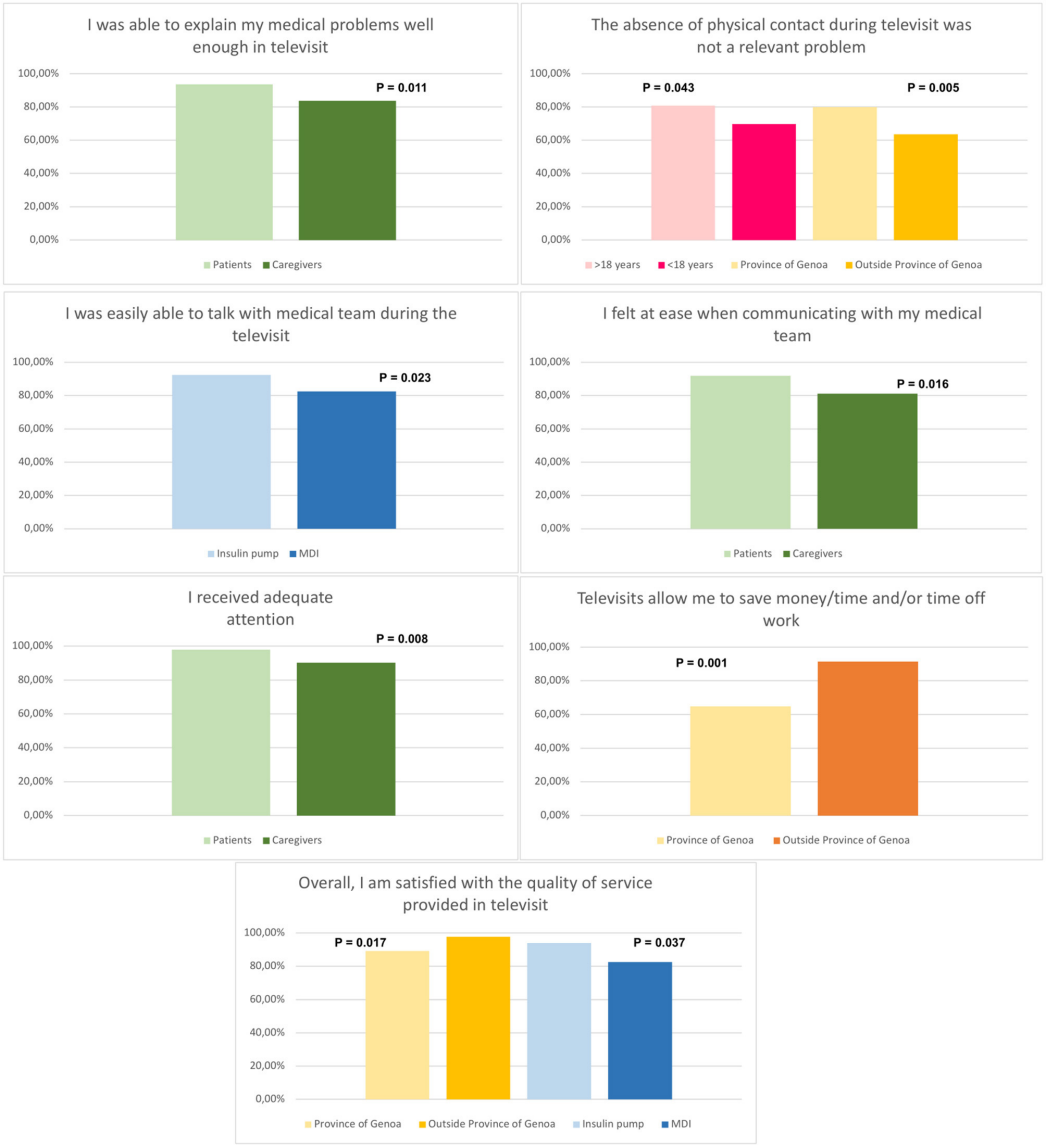
Percentage of responders who assigned neutral (0–6) and high (≥7) scores for each question.

In the part of the questionnaire relating to satisfaction in the use of telemedicine, most patients and caregivers assigned scores between 7 and 10 (good-great) in all the Clusters (Figure 1 and Table 1).

### Cluster A—Adequacy of Medical Care

#### I Was Able to Explain My Medical Problems Well-Enough via Televisit

Most individuals felt they were able to explain their medical problems well during televisits (89.9% score 7–10). We compared the satisfaction between patients (93.5%) and parents/caregivers (83.6%); both categories were able to express their medical problems, but this perception was statistically significantly better in patients than in caregivers (*p* = 0.011).

#### The Absence of Physical Contact During the Televisit Was Not a Relevant Problem

The satisfaction decreases slightly when patients were asked if the absence of physical contact was a relevant problem. The absence of physical contact was not a relevant problem for most of the participants (74.1% score 7–10) but it was less important for the population >18 years of age (19.3% score 0–6) than to those under 18 years of age (30.3% score 0–6) (*p* = 0.043). Furthermore, those who live outside the province of Genoa (63.5% score 7–10) consider the absence of physical contact a lesser problem than those who live in the province of Genoa (79.9% score 7–10) (*p* = 0.005).

#### Overall, I Am Satisfied With the Quality of the Service Provided via Televisit

92.4% of the population was overall highly satisfied with the quality of the service provided. The satisfaction for those residing outside the province of Genoa was much higher (97.7 vs. 89.1% score 0–7, *p* = 0.017). Furthermore, the gratification was found to be stronger in those treated with an insulin pump rather than multiple daily injections (93.9 vs. 85.7% score 0–7, *p* = 0.037).

### Cluster B—Psychological Impact of Telemedicine

#### I Was Easily Able to Talk With the Medical Team During the Televisit

91.1% of the population felt they could easily talk to the diabetes medical team during the televisit (score 7–10). The satisfaction was mutual in insulin pump users (92.4%) or multiple daily injections (82.5%), but statistically significantly better in pump users (*p* = 0.023).

#### I Felt at Ease When Communicating With My Medical Team

89.8% felt psychologically comfortable when communicating with the medical team (score 7–10). When comparing the score by type of therapy, psychological comfort was significantly better in pump users (91.2 vs. 81.9% score 0–7, *p* = 0.016).

#### I Received Adequate Attention

94.9% were extremely satisfied stating that they received adequate attention during telemedicine follow-up visits (score 7–10). When comparing the responses provided by patients (97.9%) and parents/caregivers (90.2%), both felt they were receiving the right amount of attention, but this perception was statistically significantly better in patients than in caregivers (*p* = 0.008).

#### I Perceived Telemedicine as an Attention Toward Me in This Period

The level of satisfaction was very high when patients were asked whether televisits were perceived as an extra attention during the pandemic period (85.8 % from 7 to 10).

### Cluster C—Possible Advantages and Future Use of Telemedicine

#### I Think That Televisits Are an Adequate Modality of Assistance for My Disease

The satisfaction decreases slightly when patients were asked whether telemedicine is an adequate type of diabetes care (68.1% from 7 to 10). There were no significant differences in the comparison of clinical and personal characteristics between participants.

#### I Am Willing to Continue Some of My Follow-Up Visits via Videocall, Keeping Appointments in Person at Longer Intervals

The gratification of families is highlighted by the fact that 80.1% of patients would happily (score 7–10) continue to be followed via telemedicine, deferring appointments in person over time.

#### Televisits Allow Me to Save Time/Money and/or Time off Work (Only for Parents)

77.0% of the population strongly agree (score 7–10) that telemedicine allows families to save money/time and/or permission to take time off work. This item was reserved for parents only and the economic question was considered more problematic in those living outside province of Genoa (91.5 vs. 64.9% score 7–10, *p* = 0.001).

### Cluster D—Telenursing

One hundred and twelve patients (41.95% of the study population) placed/changed insulin pumps after February 2020 and 36 (32.1%) of these patients changed their first set via telenursing: all of these subjects (100%) considered essential and effective the nurse's role; only 8 patients (22.2%) would have desired further assistance.

**Figure 2:**
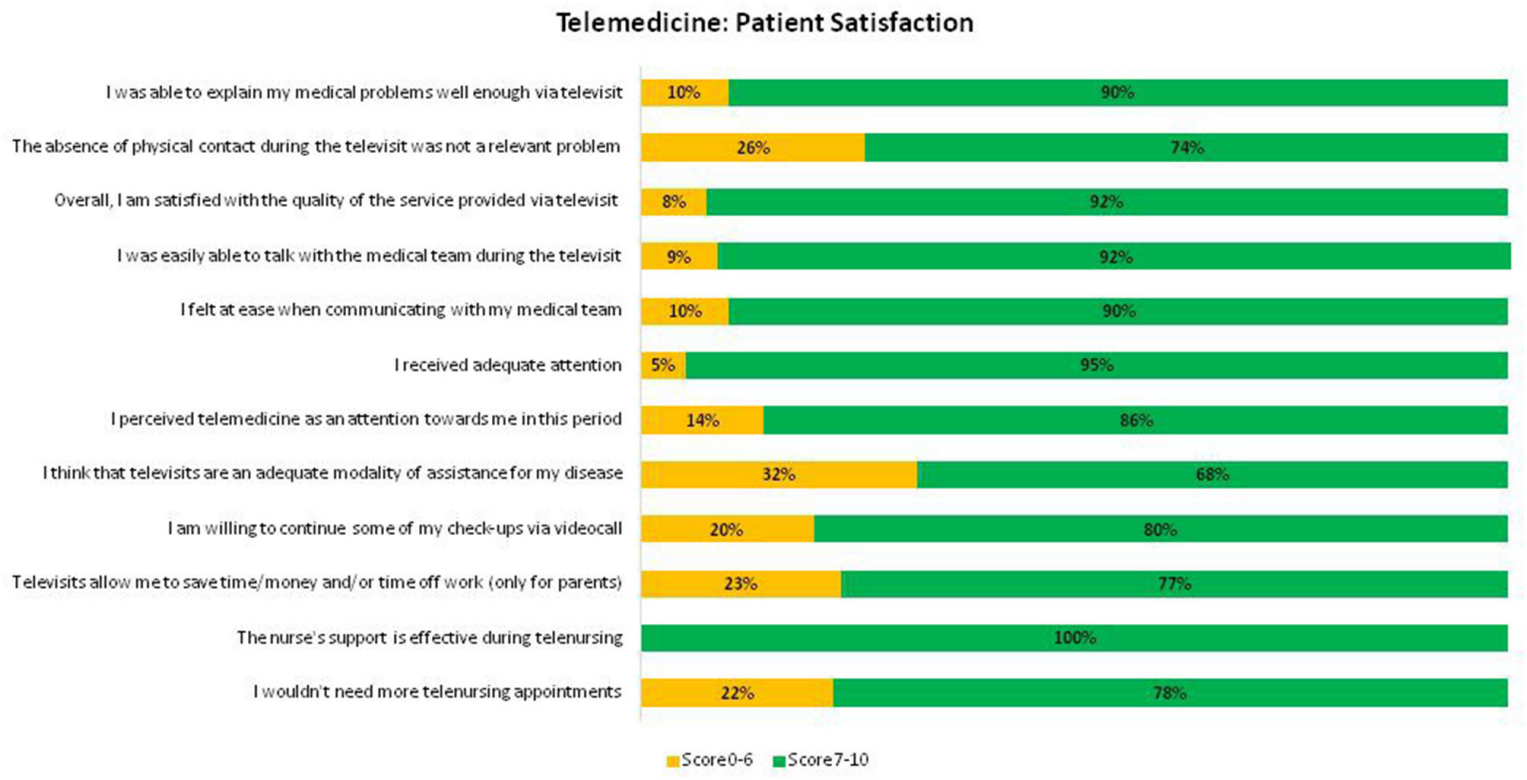
Percentage of patients who were assigned high (≥7) scores according to main patient and disease characteristics (only significant differences reported).

In Figure 2 we highlighted the significant differences in comparing responses based on patient age, participant type (parent/caregiver or patient) and based on personal or clinical characteristics reported in Table 2.

## Discussion

Our study aimed to focus attention on the pediatric and young adult population with Type 1 diabetes, evaluating the satisfaction of patients and their families in the use of telemedicine always in video-call mode both for follow-up visits and telenursing.

Data showed that both patients and parents/caregivers were able to express their medical problems, speak easily with the diabetes medical team, but most importantly they all felt comfortable during the televisits. In addition, patients and parents perceived they received adequate attention from healthcare professionals and that they had attention and support during the pandemic. All participants were overall satisfied with the telemedicine service provided by our Pediatric Diabetes Center. Comparing the answers to the questionnaire on the basis of the personal and clinical characteristics of the patients we detected some interesting aspects.

First, patients appear to be able to better explain their problems during the televisits than their parents, and they perceive more attention from the healthcare professionals than the caregivers. We suppose that this difference arises because T1D patients know themselves very well and are able to express their problems better than parents. Our Regional Pediatric Diabetes Center is located in the city of Genoa, in the center of Liguria. The cities on the far borders of the region are up to approximately 185 km from our center, making routine follow-up visits an important commitment in terms of time and money for those living outside the province. After the Morandi motorway bridge (connecting the west and east region as the only way) collapse in 2018, the regional road network suffered dramatic delays. This seems to us a sufficient reason to justify the lower perception of the lack of physical contact and the high level of patient satisfaction (97.7%) for the service in those who live further away. These same territorial factors have been considered as strengths of telemedicine in other narrative reviews that have assessed patient satisfaction with virtual healthcare (18, 22).

Although the lack of physical contact is not generally perceived as a problem, it seems to have a greater impact on patients under the age of 18 years, probably due to the confidential relationship between the healthcare team and the patient with chronic disease especially during childhood and adolescence.

Patients with insulin pumps were more able to explain and feel more comfortable communicating with healthcare professionals and were more satisfied of the quality of the service than those who are treated with multiple daily injections. Having an insulin pump with the option to download and share clinical data, inevitably leads to better communication between patient and healthcare team. Chan et al.'s review on virtual care used for glycemic management in people with diabetes highlights that patients felt motivated and had a higher sense of security considering that their health care professional could constantly monitor their data (22). As for the future perspectives in the use of telemedicine, a total of 80.1% of patients and caregivers affirm that, they would continue to use the virtual mode by keeping appointments in person at longer intervals. Currently, our center is continuing to provide regular outpatient services via telemedicine (over 50% of outpatient visits in video-call mode in 2021 out of 1,800 total visits) establishing a correct balance between patient preferences and clinical needs. Furthermore, thanks to the support of the regional Association for families of T1D patients (ADG Genova Onlus), our center is implementing the use of technology on a large scale, providing free technological devices to families who are not economically able to buy them independently; this initiative makes it possible to guarantee everyone to take advantage of telemedicine and all the advanced technological tools available for T1D therapy without discrimination.

Despite the difficult period due to the Covid pandemic, 112 patients (41.9% of participants in the study) have placed or changed an insulin pump from February 2020 to January 2022. Some of these patients underwent their first set change in video-call mode with the support of a nurse. During the telenursing appointment, both patients and parents were educated and informed about practical issues related to the correct set change, the prevention of complications and how to recognize them. Telenursing proved to be extremely useful and fundamental. Only a minority of patients would have desired support for the next pump infusion set change, demonstrating the effectiveness of telenursing in educating the patient to implement such procedures.

Previous studies have shown the fundamental role and the effectiveness of telemedicine use in improving patient glycemic control during Covid-19 pandemic (23–28). Several studies that investigate the satisfaction of the pediatric population in the use of telemedicine have recently been published (13–17), but only a few have evaluated satisfaction in patients with Type 1 diabetes.

Between March and May of 2020 Scott et al. administered an anonymous questionnaire to 7477 patients in 89 countries to assess the perception of telemedicine in people living with T1D and the results showed a great satisfaction of the service by the patients 28 (19). In May 2021 Scott et al. re-administered the same questionnaire to patients and compared the results with the previous study. There has been a remarkable drop in the proportion of patients who declared willingness to continue with remote appointments beyond the pandemic time (20). These two very interesting works mostly concern adult T1D patients and most of them carried out telemedicine in a phone call mode.

A limitation of our study is that we have omitted some fundamental aspects related to the use of IT tools in the questionnaire (device connectivity and data download). Accessing patient data is often complicated as families find it difficult to share data from insulin pumps or glycemic sensors and need specific instruction. The ability to view data as well as to make quality video-calls due to connectivity depended on good Internet connection by both the hospital service and the patient (27). It would be useful to include some questions related to satisfaction with these aspects in any future studies. Furthermore, the restriction of the survey to a cohort of T1D patients followed by a single center and mainly from the Liguria region limits the generizability of the results. Our study shows high patient and parental satisfaction, probably also related to the efficacy of the new technological devices and to the correct management of diabetes, which was not limited by the follow-up via televisits (29, 30). The strength of our study is that, to our knowledge, it is the first survey focused on patient satisfaction of telemedicine in a pediatric and young population with type 1 diabetes.

In conclusion, telemedicine and telenursing have a positive impact on the daily life of patients with Type 1 diabetes and their parents. Overall, the data collected show excellent satisfaction of the quality of the service provided and most patients prefer to continue regular follow-up visits, possibly in video-call. The most satisfied patients with the telemedicine service are pump users and residents furthest from the center. Telenursing has been an effective and appreciated tool to provide education and support to the patient in the management of practical skills relating to the use of insulin pumps and sensors.

## Data Availability Statement

The original contributions presented in the study are included in the article/supplementary material. Further inquiries can be directed to the corresponding author.

## Author Contributions

MB conceptualized the study and wrote the manuscript. MS wrote the manuscript. SP made statistical analysis. SL provided IT support. FR and CR researched data. Gd'A revised the manuscript. NM conceptualized the study, reviewed the manuscript, and contributed to discussion. MM reviewed the manuscript and contributed to discussion. All authors have read and agreed to the published version of the manuscript.

## Conflict of Interest

The authors declare that the research was conducted in the absence of any commercial or financial relationships that could be construed as a potential conflict of interest.

## Publisher's Note

All claims expressed in this article are solely those of the authors and do not necessarily represent those of their affiliated organizations, or those of the publisher, the editors and the reviewers. Any product that may be evaluated in this article, or claim that may be made by its manufacturer, is not guaranteed or endorsed by the publisher.
